# Health related quality of life in Middle Eastern children with beta-thalassemia

**DOI:** 10.1186/1471-2326-12-6

**Published:** 2012-06-22

**Authors:** Giovanni Caocci, Fabio Efficace, Francesca Ciotti, Maria Grazia Roncarolo, Adriana Vacca, Eugenia Piras, Roberto Littera, Raji Suleiman Dawood Markous, Gary Stephen Collins, Fabio Ciceri, Franco Mandelli, Sarah Marktel, Giorgio La Nasa

**Affiliations:** 1 Centro Trapianti di Midollo Osseo, P.O. “R. Binaghi”, Via Is Guadazzonis, 3, 09126, Cagliari, Italy; 2 Health Outcomes Research Unit, Italian Group for Adult Hematologic Diseases (GIMEMA) Data Center, Rome, Italy; 3 Pediatric Immuno-Hematology and BMT Unit, IRSS San Raffaele Hospital, Milan, Italy; 4 Regional Organ Transplantation Center, R. Binaghi Hospital, Cagliari, Italy; 5 Thalassemia Center, Hevi Pediatric Hospital, Duhok, Iraq; 6 Centre for Statistics in Medicine, University of Oxford, Oxford, UK; 7 Department of Hematology, University of Cagliari, Cagliari, Italy

**Keywords:** Quality of life, Thalassemia, PEDsQL 4.0

## Abstract

**Background:**

Thalassemia is a common disorder worldwide with a predominant incidence in Mediterranean countries, North Africa, the Middle East, India, Central Asia, and Southeast Asia. Whilst substantial progress has been made towards the improvement of Health related quality of life (HRQoL) in western countries, scarce evidence-based data exists on HRQol of thalassemia children and adolescents living in developing countries.

**Methods:**

We studied 60 thalassemia children from Middle Eastern countries with a median age of 10 years (range 5 to 17 years). HRQoL was assessed with the Pediatric Quality of Life Inventory (PedsQL) 4.0. The Questionnaire was completed at baseline by all patients and their parents. The agreement between child-self and parent-proxy HRQoL reports and the relationship between HRQoL profiles and socio-demographic and clinical factors were investigated.

**Results:**

The scores of parents were generally lower than those of their children for Emotional Functioning (mean 75 vs 85; p = 0.002), Psychosocial Health Summary (mean 70.3 vs 79.1; p = 0.015) and the Total Summary Score (mean 74.3 vs 77.7 p = 0.047). HRQoL was not associated with ferritin levels, hepatomegaly or frequency of transfusions or iron chelation therapy. Multivariate analysis showed that a delayed start of iron chelation had a negative impact on total PedsQL scores of both children (p = 0.046) and their parents (p = 0.007).

**Conclusions:**

The PedsQL 4.0 is a useful tool for the measurement of HRQoL in pediatric thalassemia patients. This study shows that delayed start of iron chelation has a negative impact on children’s HRQoL.

## Background

Thalassemia is an autosomal recessive congenital disease originating from countries of the Mediterranean region. Deficiencies in globin chain synthesis may lead to severe anemia requiring regular blood transfusions and iron chelation therapy starting at an early age. Despite the advances made in treatment over the past decades, many patients with beta-thalassemia major, especially those living in developing countries, do not have access to conventional and/or innovative treatment approaches. The introduction of new drugs capable of reducing the accumulation of iron in body organs, particularly the heart, liver and pancreas, has dramatically improved survival rates [1,2]. But although the number of living patients continues to rise, numerous challenges remain, such as endocrine problems, a high frequency of chronic hepatitis C as well as psychosocial morbidity associated with chronic disease [3]. Poor compliance with iron chelation therapy is another prominent unsolved problem which, today, mostly arises from the unavailability of oral iron-chelators.

Beta thalassemia major is a serious life-limiting and potentially life-threatening condition that causes substantial disruption in education and social activities. Children often need to miss school because of hospital appointments or admissions for regular monthly blood transfusion and/or treatment of complications. Their self identity is compromised and they become increasingly dependent upon others [4]. Patients and their families are conscious of the disabling nature of the condition and chronic therapy is a constant reminder that they are “*different*”, making it practically impossible to lead a normal life. Overall, the psychosocial burden affects many aspects of the patient’s life such as education, free-time, physical activities, skills, capabilities and family adjustment, the effects of which often result in anxiety, isolation and depression.

Health Related Quality of Life (HRQoL) is generally conceptualized as a multidimensional construct referring to patients’ perceptions of the impact of disease and treatment on their physical, psychological and social functioning and well-being [5,6] Several studies have investigated HRQoL in thalassemia patients but the different types of questionnaires used in the past makes it difficult to perform direct comparisons of the outcomes [7-10]. A major breakthrough came in 2003 when Varni and colleagues created the multidimentional PedsQL 4.0 questionnaire to measure the essential core domains for pediatric HRQoL: Physical Functioning, Emotional Functioning and Social Functioning, as delineated by the World Health Organization (WHO), as well as School Functioning [11,12]. The child self and parent proxy-reports of the PedsQL 4.0 measurement model are sensitive to cognitive development and contain appropriate forms for children and their parents. Some recent papers have used PedsQL 4.0 to assess HRQoL in thalassemia patients from developing countries [13-18].

However, given the paucity of research in this area and the need to rely on additional evidence-based data to further improve patient care, we conducted a study to investigate the sociodemographic and clinical factors associated with HRQoL of thalassemia children living in Middle Eastern countries. Considering that previous studies of childhood illness have shown that parents’ ratings of their child’s HRQoL tend to be lower and possibly reflect parental distress [19,20], we also evaluated the differences between HRQoL reports of the children and their parent-proxies.

## Methods

### Patients and features

From November 2007 to August 2008, we performed a cross-sectional study on a group of 60 thalassemia children and their parents, all originally coming from countries of the Middle East (i.e. Kurdistan, Palestine, Libya, Iraq and Syria). Thirty patients came to Italy to receive hematopoietic stem cell transplantation as part of a larger humanitarian project involving a knowledge exchange program with the local doctors. Only patients under the age of 18 years with a genetically confirmed diagnosis of beta-thalassemia were included in the study. The data collected both in Italy and the Middle-East were sent to the Italian Hematology Centers in Milan (San Raffaele Hospital) and Cagliari (R. Binaghi Hospital) for statistical analysis and evaluation. Questionnaires were administered in the Thalassemia Center of the Hevi Pediatric Hospital (Duhok, Iraq) immediately before transfusing patients and at the Binaghi Hospital (Italy) and San Raffaele Hospital (Italy) one month before bone marrow transplantation. Written informed consent was provided by the patients’ parents according to the declaration of Helsinki. The participants were fully informed and aware of the research objectives. The study was approved by the local ethics committee in Cagliari (Authorization n° 58/2006).

### Health related quality of life evaluation

HRQoL was assessed using the PedsQL 4.0 Generic Core Scales. This 23-item multidimentional model encompasses the essential core domains for pediatric HRQoL measurement: Physical Functioning (8 items), Emotional Functioning (5 items) and Social Functioning (5 items), as delineated by the World Health Organization (WHO), as well as School Functioning (5 items) [11,12]. Additionally, the PedsQL 4.0 questionnaire contains a Psychosocial Health Summary Score which represents the sum of items over the number of items answered in the Emotional, Social and School functioning scales [21]. To create the total PedsQL score, the mean is computed as the sum of all items over the number of items answered on all scales. The child self and parent proxy-reports of the PedsQL measurement model are sensitive to cognitive development and contain appropriate forms for children aged 5–7, 8–12 and 13–18 years and their parents. The reliability, internal consistency and validity of the PedsQL questionnaire have been assessed in pediatric populations with acute and chronic health conditions, besides in physically healthy pediatric populations [11,12]. Each item is rated on a 5-point Likert scale from 0 (Never) to 4 (Almost always) and on a 3-point scale for young children (5–7 years): 0 (Not at all), 2 (Sometimes) and 4 (A lot). The scores for each dimension are calculated as follows: the mean score is represented by the sum of the items over the number of items answered; missing values are replaced by the mean score of the remaining items; if more than 50% of the items in a given scale are missing, the scale scores are not computed. Raw scores are transformed into standardized scores on a scale from 0 to 100 with higher scores representing higher functioning levels.

For logistic reasons, the PedsQL 4.0 Generic Core Scales were administered both in homeland countries (50%) and in Italy (50%). The arabic version of this questionnaire was validated by Garaibhe et al. in 2011 on a cohort of 128 Jordan thalassemia patients [18] (Table 1). Our cohort of patients and parents all spoke the Arabic language. Questionnaires in the Arabic language were completed independently by children above 8 years and their parents. Children aged 5–7 years were questioned by trained interviewers.

**Table 1:** Overview of studies evaluating HRQoL with PEDsQL in pediatric patients with thalassemia. NR = not reported

**Study**	**Sample and nationality**	**Physical functioning mean (SD)**		**Emotional functioning mean (SD)**		**Social functioning mean (SD)**		**School functioning mean (SD)**		**Psychosocial summary score mean (SD)**		**Total summary score mean (SD)**	
		Patients	Parents	Patients	Parents	Patients	Parents	Patients	Parents	Patients	Parents	Patients	Parents
Ismail et al. 2006 [13]	78 Malaysia	69.1 (16.4)	NR	68.1 (17.2)	NR	74.3 (18.7)	NR	60.1 (16.4)	NR	67.58 (12.8)	NR	68.9 (12.1)	NR
Cheuk et al. 2008 [14]	25 Hong Kong	66.7 (16.0)	NR	60.8 (17.1)	NR	82.5 (17.7)	NR	75.4 (19.5)	NR	72.9 (13.6)	NR	NR	NR
Clarke et al. 2009 [15]	22 UK	NR	66.5 (21.8)	NR	73.6 (22.7)	NR	77.6 (19.9)	NR	60.9 (27.5)	NR	68.3 (19.7)	NR	69.1 (18.1)
Thavorncharoensap et al. 2010 [16]	315 Thailand	78.2 (14.8)	NR	75.9 (16.6)	NR	83.7 (14.7)	NR	67.9 (15.9)	NR	75.5 (12.8)	NR	76.7 (11.4)	NR
Surapolchai et al. 2010 [17]	54 Thailand	79.6 (2.3)	72.2 (2.6)	NR	NR	NR	NR	NR	NR	77.4 (2.3)	74.6 (2.2)	78.5 (2.1)	73.4 (2.2)
Garaibeh et al. 2011 [18]	128 Jordan	54.2 (15.1)	NR	62.4 (23.3)	NR	73.3 (20.9)	NR	46.7 (21.1)	NR	NR	NR	59.2 (16.3)	NR
This report 2012	60 Middle East	68,4 (27,2)	67,1 (28,0)	76,9 (24,6)	71,7 (25,3)	76,4 (20,6)	73,9 (23,2)	69,4 (21,4)	67,9 (21,4)	73,6 (20,0)	69,6 (20,2)	71,9 (20,6)	69,3 (19,9)

### Statistical analysis

The clinical and demographic features of the patients were presented in terms of percentages, median, mean, standard deviation and range. Descriptive measures for PedsQL domains were reported as median and inter-quartile ranges. Because of the non-normality of responses, differences between patient and parent responses on the PedsQL 4.0 were analyzed using the paired Wilcoxon signed rank test. The differences were expressed as (pseudo) median with 95% confidence intervals. Different responses of boys and girls to items within a domain, as well as ferritin levels above 1300 mg/dl (Yes/No), Hepatomegaly (Yes/No), transfusion and iron chelation frequency (regular/slightly irregular/irregular), were analyzed using the Mann–Whitney test. Cut-off points were based on standard clinical guidelines. Ferritin values above 1300 μg/dl clearly suggest liver iron overload (Table 2).

**Table 2:** Clinical and socio-demographic characteristics of 60 thalassemia children

**Characteristic**	**n (%)**
Age; median (range)	10.0 (5 to 17) years
Sex	
Male	34 (57)
Female	26 (43)
Country of origin	
Kurdistan	10 (17)
Libya	8 (13)
Palestine	1 (2)
Syria	10 (17)
Iraq	31 (52)
Age at diagnosis in months; median (range)	8 (1 to 48)
Age at first transfusion in months; median (range)	11.5 (2 to 60)
Age at start of iron chelation in months; median (range)	49 (12 to 120)
Transfusion frequency	
Regular (pre-transfusion hemoglobin ≥ 9gr/dl)	13 (22)
Fairly irregular (pre-transfusion hemoglobin ≥ 7.5 and<9 gr/dl)	34 (57)
Irregular (Pre-transfusion hemoglobin <7.5 gr/dl or frequent transfusion reactions)	13 (22)
Hepatomatology > 2 cm	
Yes	46 (77)
No	13 (22)
Unknown	1 (2)
Hepatitis	
Yes	39 (65)
No	21 (35)
Ferritin >1300 ng/ml	
Yes	47 (78)
No	13 (22)
Iron chelation	
Regular administration (once-daily)	13 (22)
Slightly irregular administration (at least once a week)	29 (48)
Irregular administration (less than once a week or none)	16 (27)
None	2 (3)

Association between patient characteristics and the total PedsQL score was analyzed by univariate linear regression. Multivariable linear regression models using the PedsQL score as the outcome (which was normally distributed) and the patient characteristics as predictors were constructed using Bayesian model averaging [22].

## Results

The 60 children and parents invited to participate in the study completed the PedsQL questionnaire with a response rate of 100%. Patient clinical and socio-demographic characteristics are shown in Table 2. Despite the fact that beta-thalassemia major was diagnosed at a median age of 8 months and that blood transfusions were started at a median age of 11.5 months, there was a significant delay in the start of iron chelation therapy, reaching a median of 49 months (range 12–120) after birth. Only 22% of the children had been offered regular transfusion support (pre-transfusion hemoglobin value ≥ 9gr/dl) and 78% of the patients had either received irregular iron chelation therapy (less than once a week) or none at all. Seventy-seven percent of the patients had hepatomegaly which in most cases (65%) was due to coexisting chronic hepatitis B or C; 78% of the patients had ferritin values above 1300 μg/dl (Table 2).

### HRQoL based on clinical and sociodemographic characteristics

No statistically significant differences were found for any of the PedsQL domains after categorizing patients according to ferritin levels, hepatomegaly, frequency of transfusions and iron chelation therapy. A borderline difference (p = 0.047) was found for gender within the PedsQL School domain. Girls had higher median scores than boys: 80 (range, 65.4 to 90) and 70 (range, 45 to 80), respectively.

Further analysis was performed to investigate the global child and parent ratings for well-being (total PedsQL score) and the association with clinical and demographic features. Univariate and multivariate analysis showed that a delayed start of iron chelation had a negative impact on total PedsQL scores both in children (p = 0.046) and parent reports (p = 0.007). No significant association was found with any of the other factors considered (sex, age at diagnosis, age at first transfusion, transfusion frequency, hepatomegaly, hepatitis, ferritin above 1300 mg/dl, frequency of iron chelation) (Table 3). No differences were observed between patients who received supportive care in the Middle East and those treated in Italy before transplantation.

**Table 3:** Univariate and multivariable linear regression analyses of association between clinical and demographic characteristics and total PEDsQL scores in thalassemia patients and parents

**Characteristic**	**Total PEDSQL score (patients)**		**Total PEDSQL score (parents)**	
	Univariate*β* (95% CI)	Multivariable*β* (95% CI)	Univariate*β* (95% CI)	Multivariable*β* (95% CI)
Sex	1.49 (95% CI −9.09 to 12.07) p = 0.783	-	4.40 (95% CI −5.81 to 14.61) p = 0.401	-
Age at diagnosis	0.22 (95% CI −0.20 to 0.63) p = 0.311	-	−0.01 (95% CI −0.42 to 0.39) p = 0.959	-
Age at first transfusion	0.15 (95% CI −0.22 to 0.53) p = 0.423	-	−0.12 (95% CI −0.48 to 0.25) p = 0.528	-
Age at start of iron chelation	−0.17 (95% CI −0.33 to 0) p = 0.046	−0.17 (95% CI −0.33 to 0) p = 0.046	−0.21(95% CI −0.36 to –0.06) p = 0.007	−0.21(95% CI −0.36 to –0.06) p = 0.007
Transfusion Frequency	7.04 (95% CI −5.56 to 19.65) p = 0.278	-	3.30 (95% CI −9.03 to 15.63) p = 0.602	-
Hepatomegaly	1.46 (95% CI −11.41 to 14.32) p = 0.278	-	5.11 (95% CI −7.29 to 17.51) p = 0.423	-
Hepatitis	−2.91 (95% CI −13.88 to 8.06) p = 0.605	-	−2.21 (95% CI 12.87 to 8.45) p = 0.686	-
Ferritin (>1300)	10.77 (95% CI −1.66 to 23.20) p = 0.095	-	3.05 (95% CI −9.28 to 15.38) p = 0.629	
Iron Chelation	6.24 (95% CI −6.40 to 18.87) p = 0.337	-	10.95 (95% CI −1.08 to 22.98) p = 0.080	-

### Child self and parent proxy-reports of HRQoL

Table 4 reports the differences in child and parent PedsQL domain scores, expressed as medians and inter-quartile ranges. When comparing the level of agreement between child self and parent proxy-ratings, it was found that parents tended to slightly underestimate their child’s HRQoL. The scores of parents were lower for Emotional Functioning (median 75 vs 85; p = 0.002), Psychosocial Health Summary (median 70.3 vs 79.1; p = 0.015) and the Total Summary Score (median 74.3 vs 77.7 p = 0.047) (Table 4). Multivariate analysis did not reveal any associations with age or gender in either patients or their parents.

**Table 4:** Differences in child and parent PedsQL domain scores, expressed as median and inter-quartile ranges

**PEDsQL scale**	**Patients**	**Parents**	**Difference (95% CI)**	**P value**
Physical Functioning	75 (56.3, 91.40)	75 (55.5, 85.18)	3.75 (−1.55, 7.8)	0.112
Emotional Functioning	85 (60, 100)	75 (50, 95)	10 (2.5, 15)	0.002*
Social Functioning	82.50 (60, 91.25)	75.8 (65, 90)	0.74 (−3.5, 7.5)	0.576
School Functioning	75.00 (50, 85)	70 (50, 80)	2.5 (−5, 7.5)	0.465
Psychosocial Health Summary	79.15 (61.88, 88.30)	70.3 (59.58, 83.72)	4.2 (0.85, 7.5)	0.015*
Total Summary Score	77.75 (61.45, 88.28)	74.35 (60.35, 82.77)	2.45 (4.62, 4.9)	0.047*

## Discussion

Pediatric assessment of HRQoL has traditionally been based on reports compiled by parents, which may not reflect the child’s perceptions. On this ground, we investigated both the parents’ perceptions and the child’s perceptions of disease and treatment-related burden. The value of combining child self and parent proxy-reports to investigate health, functioning, abilities and emotions in children, is increasingly recognized [20].

The main evidence emerging from this cross-sectional analysis was that a delayed start of iron chelation was independently associated with a negative impact on the total PedsQL score (Table 3). Interestingly, this independent association was confirmed by both child self and parent proxy-reports. Delayed iron chelation can lead to excessive accumulation of iron in body organs. Health issues arise especially when excess iron is stored in the heart, liver or pancreas.

This finding underlines the need for early and appropriate scheduling of routine iron chelation therapy in these patients. The introduction of promising new oral iron chelators has significantly improved depletion of iron stores in patients, with a major impact on well-being and survival [1,2]. However, in developing countries the cost of iron chelation is either too expensive or not available and iron overload in pediatric thalassemia patients is a common finding.

When we investigated the differences between PedsQL median scores of children and their parents, we found that parent scores were lower for Emotional Functioning, Psychosocial Health Summary and the Total Summary Score (Table 4). It would appear that parents tend to consider HRQoL of their children more compromised in domains dealing with interpersonal relationships, rather than those concerning physical impairment. A possible explanation may be that parents of thalassemia children unconsciously project their pessimistic feelings onto their child’s functioning. Creemens et al. [20] speculated that several factors influenced the agreement between child self and parent proxy-reports using the PedsQL generic core scales. They retain that much depends upon the domain being measured, with higher agreement for the physical aspects of health compared to emotional or social aspects. Furthermore, they postulated that in the healthy population, agreement between child and parent proxy-reports of child HRQoL may be affected by the child’s age and the parents’ own HRQoL. In contrast with our findings, the only previous study of thalassemia children to assess both patient and the parent perspectives, showed a good level of agreement between child and parent reports [17]. Hence, further research will be required to explore whether parent–child agreement is dependent on additional factors, such as the parent-to-child relationship (mother vs father) or different types of disease.

Only a few studies have used PedsQL 4.0 to assess HRQoL in thalassemia patients. Details are given in Table 1. Overall, PedsQL domain scores were collected from 600 patients and 76 parents. Ismail et al. [13] reported the data obtained on a cohort of 78 Malaysian patients (mean age 11.9 years) and compared the scores to those of 235 healthy controls. The results showed a 10% to 24% reduction in the physical, social and school functioning domains of the patients, regardless of age, gender, ethnicity and household income. The authors recommended that the Ministry of Health continue to support these patients with the supply of free desferal. Cheuk et al. [14] conducted a comparative study in Hong Kong to evaluate the differences in HRQoL between 25 transfusion dependent thalassemia patients and 15 patients who underwent hematopoietic stem cell transplantation. PEDsQL scores of the first group were not significantly different from those of the transplanted patients, indicating that conventionally treated patients adapt relatively well to the burden of chronic illness and treatment. Clarke et al. [15] focused on mothers’ reports of 22 thalassemia children (aged on average 10 years) waiting for hematopoietic stem cell transplantation. The results showed a significant compromise in HRQoL in spite of the fact that the children displayed behaviour comparable to that of the general population.

Thavorncharoensap et al. [16] evaluated PEDsQL scores in 315 thalassemia children and adolescents in Thailand. Again, the school functioning subscale scored the lowest, with a mean of 67.9 (69.4 in our report). Age at onset of anemia, age at first transfusion, irregular iron chelation therapy and low pre-transfusion hemoglobin levels were factors significantly affecting HRQoL. The authors suggested the introduction of suitable programs aimed at providing psychosocial support and a link between the patient, school officials, the family and the physician. Surapolchai et al. [17] conducted a study on 75 thalassemia children in Thailand and were the first to assess HRQoL from both the patients’ and parents’ perspective. Child self-reports were negatively influenced by low family income and an onset age of anemia below 2 years, whereas the negative predictor of total HRQoL score in parent proxy-reports was the frequency of red blood transfusions.

Finally, Garaibeh et al. [18] compared the outcomes of PEDsQL obtained on a sample of 128 Jordanian thalassemic children aged 8–18 years with those of 83 healthy children. The patients had significantly lower HRQoL mean scores in all dimensions compared to their healthy counterparts. The lowest mean score was reported for the school domain (46.7): healthcare providers, counsellors and school teachers have an important role in helping children to overcome this problem.

Many aspects of these previous reports underline the effectiveness of the approach applied in the present study which offers a unique perspective of HRQoL by evaluating both child-self and parent-proxy reports.

## Conclusions

Although larger studies are warranted, our results show that a delayed start of iron chelation has a negative impact on total PedsQL scores of both child and parent reports. Parent ratings of their child’s HRQoL were lower for emotional functioning, psychosocial functioning and total HRQoL, suggesting the need to enhance the understanding and support of the parents. By increasing the awareness and knowledge levels of the parents, we can help sick children in developing countries to get the best care locally and to thus improve HRQol. A limitation of the present study was that although the PedsQL questionnaire is sensitive to cognitive development, we did not perform a complete cognitive or psychological evaluation based on normative scale scores, which would have been helpful in order to avoid confounding factors (i.e. cognitive, psychological or psychiatric problems in parents or children). Another limitation of the study was that HRQoL meaurements were obtained in a cross-sectional manner. Further research with a longitudinal study design and the recruitment of a healthy control group is warranted.

Thalassaemia patients and their parents require lifelong psychological support for prevention of mental health issues. Several effective psychological strategies are available. Cognitive-Behavioural Family Therapy (CBFT) can be an effective psychological approach to children with beta-thalassaemia major, capable of increasing compliance to treatment, lessening the emotional burden of disease and improving the quality of life of caregivers [23]. Overall, child self and parent proxy-reports represent a valid methodological approach which should be highly recommended for investigation of HRQoL in pediatric patients.

## Abbreviations

HRQoL: Health related quality of life; PedsQL: Pediatric quality of life inventory.

## Competing interests

The authors declare that they have no competing interests.

## Authors’ contributions

GC, FE and GLN conceived and designed the study, acquired and analyzed the data and drafted initial and final versions of the manuscript. FC, MGR, AV, EP, RL, RSMD, FC, FM and SM acquired data and contributed to the clinical management of patients. GSC performed statistical analysis. All authors read and approved the final manuscript.

## Authors’ information

FE is Chairman and GC is Co-chairman of the GIMEMA (Italian Group for Adult Hematologic Diseases) Quality of Life Working Group.

## Pre-publication history

The pre-publication history for this paper can be accessed here:

http://www.biomedcentral.com/1471-2326/12/6/prepub
